# Assessment of socio-behavioural correlates and risk perceptions regarding anthrax disease in tribal communities of Odisha, Eastern India

**DOI:** 10.1186/s12879-022-07035-9

**Published:** 2022-01-15

**Authors:** Matrujyoti Pattnaik, Jaya Singh Kshatri, Hari Ram Choudhary, Debaprasad Parai, Jyoti Shandilya, Asit Mansingh, Arun Kumar Padhi, Sanghamitra Pati, Debdutta Bhattacharya

**Affiliations:** 1 Department of Microbiology, ICMR-Regional Medical Research Centre (Department of Health Research, Ministry of Health & Family Welfare, Government of India), Chandrasekharpur, Bhubaneswar, 751023 India; 2 Office of the Chief District Medical Officer Koraput, Department of Health and Family Welfare, Government of Odisha, Koraput, 764020 India

**Keywords:** Anthrax, Knowledge-attitude-practices, Koraput, Endemic region, One Health

## Abstract

**Background:**

This study is a baseline survey to assess the knowledge, attitude and practices with regards to the anthrax disease among the communities before demonstrating a One Health approach for elimination of human anthrax in an endemic district of Odisha. A total of 2670 respondents from 112 villages of 14 blocks were interviewed for the study using a structured questionnaire by multi-stage sampling method. Descriptive statistics were reported and logistic regression was performed to estimate the relationship between the variables and knowledge of anthrax.

**Result:**

Out of 2670 participants in the study, 76.25% were male and about half were illiterate. Most of the respondents (54.19%) were involved in agriculture as an occupation. 71% of the respondents had livestock in their houses and farming was the main purpose for keeping the livestock. Only one-fifth of the respondents (20.26%) knew about anthrax and a majority of them have come across the disease during community outbreaks. Almost 25.9% of livestock owners had knowledge about vaccination against anthrax disease although 83.4% of the livestock owners disposed the animal carcass by burial method.

**Conclusion:**

The study findings indicated that the community members had poor knowledge of cause, symptoms, transmission and prevention of anthrax disease which may be improved by a One Health approach.

**Supplementary Information:**

The online version contains supplementary material available at 10.1186/s12879-022-07035-9.

## Introduction

Anthrax is a globally neglected zoonotic disease caused by *Bacillus anthracis* and is widely found in cattle, goats, sheep and deer [1, 2]. Humans often got the disease when they come in close contact with an infected animal. The risk of an anthrax outbreak in the endemic areas is increased due to the exposure of inactive *Bacillus anthracis* spores from the soil [3, 4]. These spores are then swallowed by the herbivores during grazing which are then germinated inside the body and manifests various disease symptoms [5].

Anthrax has three clinical forms in humans based on the route of infection namely cutaneous (skin), gastrointestinal (ingestion) and pulmonary anthrax [6]. It is a disease more prevalent among animal handlers who get infected from contamination during the production, processing and handling of animal products and also among people who ingest/consume infected meat [7, 8]. Lack of awareness among livestock owners regarding anthrax is one of the most important factors leading to the disease outbreaks and becomes a big hurdle in controlling the disease [9].

In India, anthrax cases have been reported from different states like Odisha, Andhra Pradesh, Jammu & Kashmir, Tamil Nadu and Karnataka. In the last 15 years, 14 out of 30 districts have witnessed repeated outbreaks of anthrax affecting at least 1208 people, mostly cutaneous anthrax of which 436 had died in Odisha [10, 11]. The anthrax outbreaks become an endemic in few parts of this state and the most frequently affected districts are Koraput, Rayagada, Malkangiri, Sundargarh, and Kandhamal of which Koraput district tops the list with more than 300 human cases and more than 10 deaths with confirmed anthrax infection during the last 6 years [11, 12].

The following study is a baseline study which was conducted as a part of a One Health approach demonstration to map the current knowledge, attitude and practices among the communities residing in Koraput district of Odisha and to enlist the risky practices that can be a potential threat to catch the disease.

## Materials and methods

### Study design

The study was a cross-sectional survey conducted as a baseline before using the intervention of the One Health approach for the elimination of human anthrax cases in the district. This study was conducted from February 2020 to October 2020. All methods were carried out in accordance with relevant guidelines and regulations.

### Study settings

The study was undertaken in the Koraput district, which is situated in south Odisha spreading out over 8807 sq. km with 14 blocks and 2028 revenue villages. It lies between 18.8561° N latitude and 82.7347° E longitude.

### Sample size

The sample size was calculated by using the formula stated in Bhattacharya et al. [13]. Assuming the knowledge of anthrax in the community as 5% with a design effect of 1.3 and confidence interval of 95%, relative precision of 20% and non-response rate of 10% was calculated to a total of 2608 which was rounded off to 2640.

### Sampling method

A multistage simple random sampling method was adopted for selecting the study participants from all the 14 blocks in the district. Block is a district sub-division and Gram Panchayat is the basic village governing body in Indian villages consisting of several villages. A list of fourteen blocks with total Gram panchayats and their total villages were made according to census 2011 for randomisation. The list of selected Gram panchayats and villages are provided as Additional file 1 (Annexure 1 and 2).

**Fig. 1:**
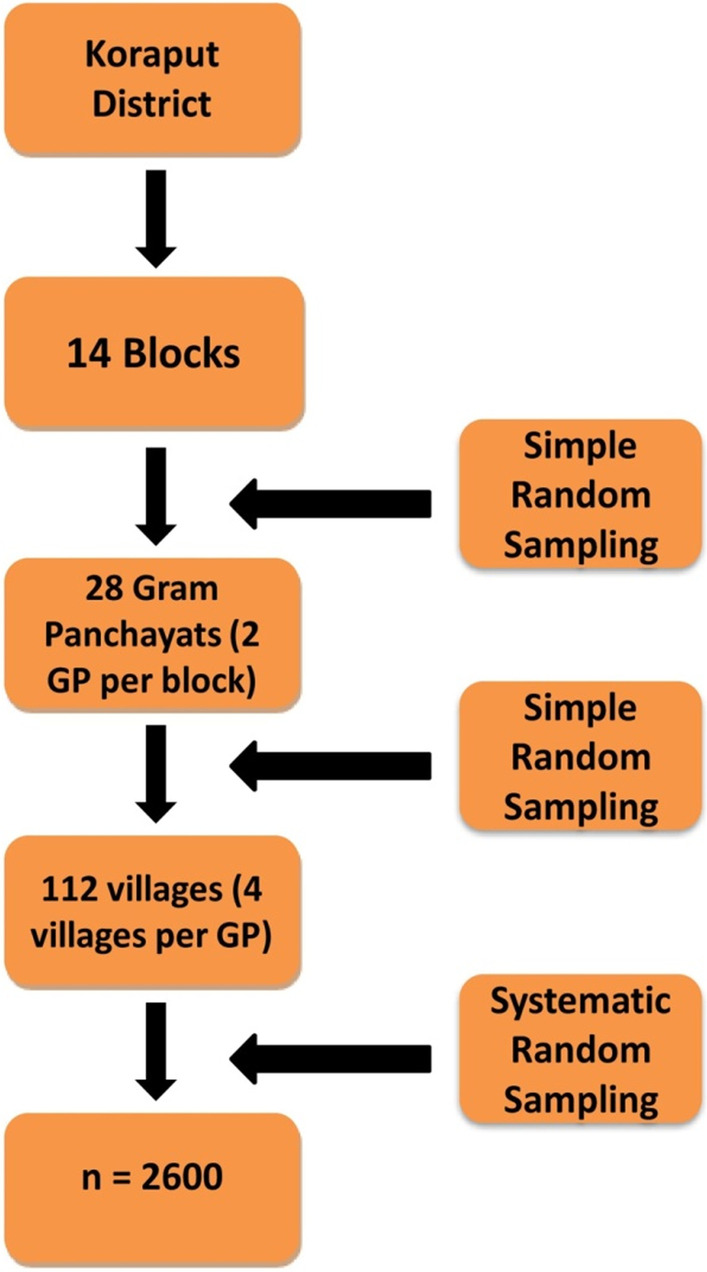
Sampling methods for the selection of study participants

We have selected two Gram Panchayats from each administratively divided block and further four villages from each Gram Panchayat were selected based on simple random sampling using the random number generation method. If the number of villages in a Gram Panchayat was less than four, then another Gram Panchayat was selected randomly from the same block. In this way, 112 villages were selected for data collection in the district. In each selected village, households were selected systematically and only one adult individual was enrolled from each selected house. If there were two or more adults in one house, then simple random sampling was followed to select an individual for the study. The flow diagram for sampling is provided in (Fig. 1).

### Data collection

A questionnaire was developed with 85-items based on the contents described in the Bhattacharya et al [13]. The structured questionnaire primarily consisted of semi-open questions and was arranged into domains namely socio-demographic characteristics (age, sex, education level, occupation, number of people in the household, animal ownership status), information on domestic animal (type of livestock, grazing habits, years of experience in handling livestock), dead-animal handling, food habits (consumption of meat and its sources) and knowledge assessment & awareness of the respondent about anthrax disease (signs or symptoms, transmission, precaution and prevention). The questionnaire was uploaded in Open Data Kit and data were collected using electronic devices. The field staff were provided with hands-on training for collecting the data through the tablet. The collected data were downloaded at regular intervals and data cleaning and monitoring were carried out by the data handler.

### Data analysis

Statistical Package for Social Sciences (SPSS) version 21 was used for data analysis. The frequency distribution and percentages of the variables were calculated. The bivariate and multivariate logistic regression determined whether variables such as age, gender, educational level, occupation, presence or absence of livestock and meat consumption influenced the knowledge of anthrax at a significance level of 0.05. Heat map regarding knowledge of anthrax disease was done using QGIS (ver. 3.10).

## Results

A total of 2670 individuals were surveyed from 112 villages across 14 blocks of the district. Socio-demographic characteristics of the study population are provided in Table 1. More than 3/4th (76.25%) of the studied population were male with a mean age of males being 40.4 ± 14.4 years and the mean age of females were recorded as 38.1 ± 13.8 years. Most of the study population were in the 18–29 age group (26.75%). The study participants mainly belonged to the Hindu religion (86.78%) and were tribal (62.43%). Respondents were classified into illiterate who received no formal education and literate who have completed atleast primary, secondary or tertiary level of education. More than 50% of the respondents were illiterate and a majority of the population (54.19%) were found to be engaged in agricultural practices.

**Table 1:** Socio-demographic information of the study participants in Koraput district

Variables	Frequency	Proportion (%)
Gender		
Male	2036	76.25
Female	634	23.75
Age-groups		
18–29	714	26.75
30–39	633	23.7
40–49	542	20.3
50–59	397	14.87
60 and above	384	14.38
Category		
General	259	9.7
Other backward caste	330	12.36
Schedule caste	414	15.51
Schedule tribe	1667	62.43
Education		
Illiterate	1432	53.63
Literate	1238	46.37
Annual income (in Rs)		
Less than 10,000	496	18.58
10,000–50,000	1598	59.85
More than 50,000	576	21.57
Household size		
1–3	533	19.96
4–6	1546	57.91
7 and more	591	22.13
Occupation		
Unemployed	128	4.79
Government service	61	2.28
Private service	87	3.27
Housewife	148	5.54
Agriculture	1447	54.19
Business	173	6.48
Daily labour	626	23.45

Information about livestock handling is provided in Table 2. Of all the households visited, 71.42% of the households were having livestock. Most of the households were having cattle/buffalos (96.06%), then goat (24.49%) and sheep (18.56%) respectively. The main purpose for keeping the livestock was for cultivating the agricultural field (82.12%) followed by dairy farming (24.59%) and selling meat (17.93%). Forest (61.98%) was the most preferred place for grazing of animals along with grass fields (18.51%) and agricultural lands (17.99%).

**Table 2:** Information about livestock and animal handling

Question	Frequency	Proportion (%)
Respondents having livestock (n = 2670)		
Yes	1907	71.42
No	763	28.58
Person’s dealing with the livestock (n = 1907)		
Myself	1269	66.54
Wife/Husband	218	11.43
Parents	222	11.65
Son	104	5.45
Other relatives	94	4.93
Respondents having which livestock^#^ (n = 1907)		
Cattle/Buffalo	1832	96.06
Goat	467	24.49
Pig	30	1.57
Sheep	354	18.56
Purpose of keeping livestock animals (n = 1907)		
Leather industry	2	0.1
Skinning	6	0.31
Dairy	469	24.59
Farming	1566	82.12
Selling meat	342	17.93
Respondent’s preferred place for grazing (n = 1907)		
Forest	1182	61.98
Agricultural land	343	17.99
Grass field	353	18.51
Buy commercial fodder	29	1.52
Respondent’s having how many years of experience in handling livestock animals (n = 1907)		
Less than 1 year	140	7.34
1–5 years	200	10.49
5–10 years	284	14.89
More than 10 years	1283	67.28

^#^Multiple choices were noted

**Fig. 2:**
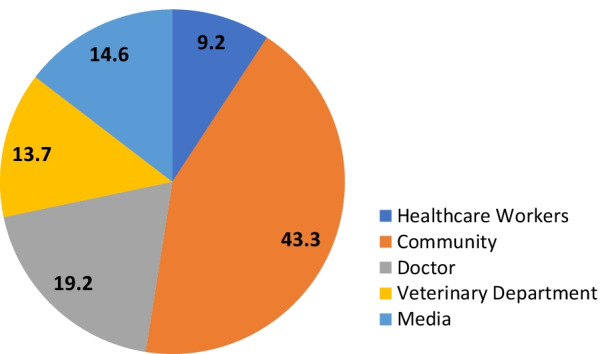
Distribution of information regarding anthrax from the participants who knew about anthrax

**Fig. 3:**
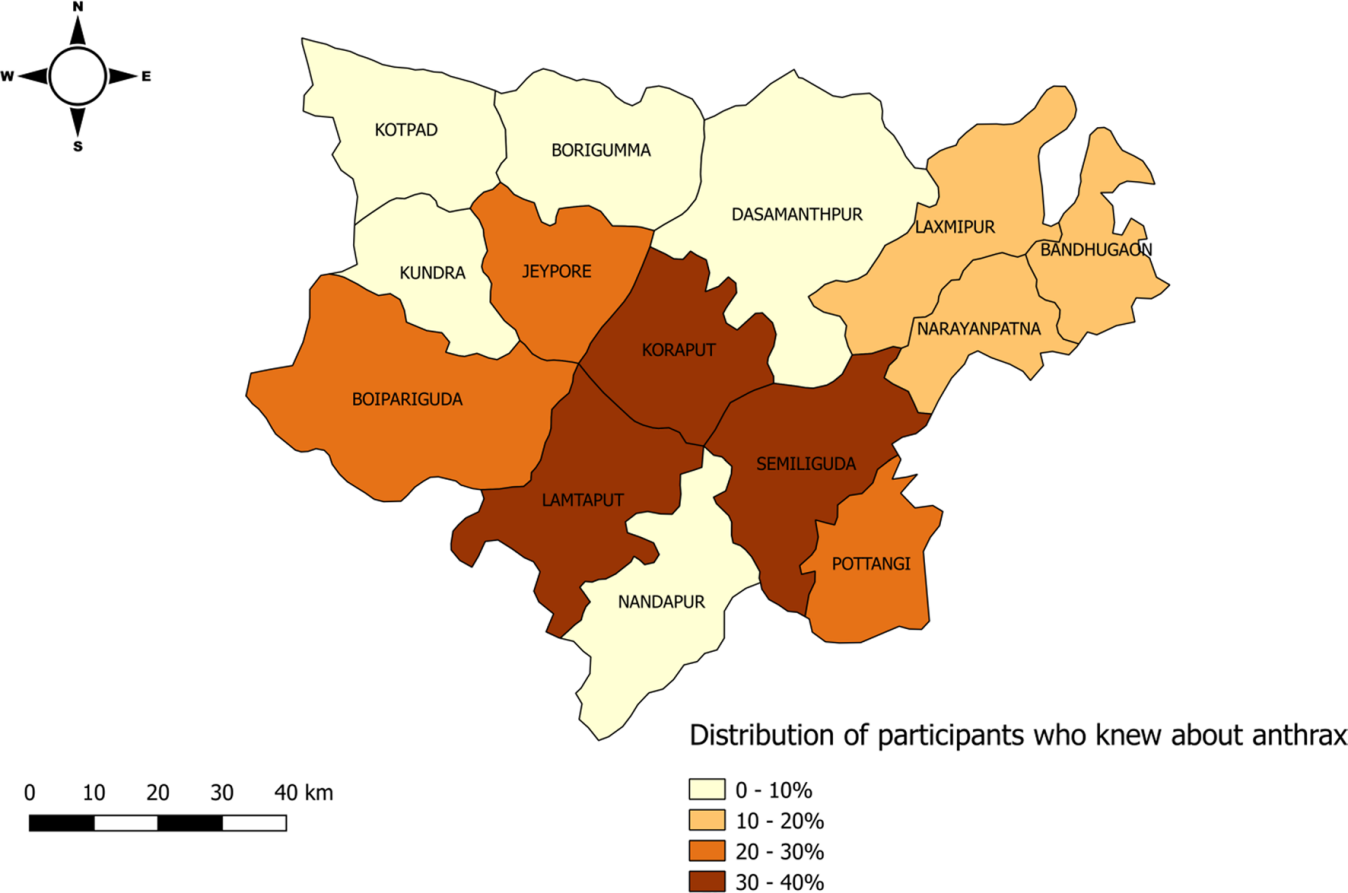
Chloropleth map showing distribution of participants who knew about anthrax across blocks of Koraput

Knowledge of anthrax refers to the people who could tell the signs and/or symptoms and/or transmission of anthrax in either humans or animals. Categorization of the study population according to the knowledge, attitude and practices regarding anthrax is shown in Table 3. Around 20.26% of people were aware of anthrax disease in animals and humans. Most of the respondents (43.3%) knew about anthrax from the community, 19.2% from the doctors, 13.7% from the veterinary department, 14.6% from media such as newspapers, internet, television, etc. and 9.2% from healthcare workers like Accredited Social Health Activist (ASHA), Auxiliary Nurse-Midwife (ANM), Anganwadi Workers (AWW), Primary Health Centre (PHC) doctors etc. (Fig. 2). A heat map for blocks regarding the distribution of participants who knew about anthrax is given in Fig. 3. One-fourth of the population handling livestock (25.9%) was aware of the anthrax vaccination of animals which is a preventive method for controlling anthrax disease among livestock animals. More than 70% of livestock owners inform relevant authorities on suspected anthrax cases in animals, whereas 23% of the people do not report it. About the risk practices and intended behaviour of persons towards anthrax, 14.75% of the study population was involved in the consumption of beef and 25.03% consume animal blood in their diet. Approximately 4.2% of the livestock owners consume the dead animal’s meat. Among livestock owners, 2.8% distribute the dead animals among villagers and 2.6% sell the carcass. Around 83% of the livestock owners were involved in burial of dead animals where they dig a land upto 5 feet and bury the dead animal in the presence of livestock inspector or veterinary doctor.

**Table 3:** Participant’s knowledge, attitude and practices towards anthrax disease in Koraput

Model	Question	Frequency	Proportion (%)	95% CI
Knowledge	Have heard about anthrax in animals or humans (n = 2670)			
	Yes	541	20.26	18.7—21.8
	No	2129	79.74	78.1—81.2
	Knowledge about symptoms of animal anthrax (n = 2670)			
	Yes	227	8.50	7.4–9.6
	No	2443	91.50	90.3–92.5
	Knowledge about transmission of anthrax in animals (n = 2670)			
	Yes	271	10.15	9.1–11.3
	No	2399	89.85	88.6–90.9
	Knowledge about symptoms of human anthrax (n = 2670)			
	Yes	296	11.08	9.9–12.3
	No	2374	88.92	87.6–90.1
	Knowledge on mode of transmission of anthrax from animal to human (n = 2670)			
	Yes	207	7.75	6.7–8.8
	No	2463	92.25	91.1–93.2
	Knowledge about preventive method of anthrax—vaccination of livestock (n = 1907)			
	Yes	495	25.96	24.0–27.9
	No	1412	74.04	72.0–75.9
	Information about financial help from the government for the burial of dead animals (n = 1907)			
	Yes	22	1.15	0.7–1.8
	No	1885	98.85	98.2–99.3
Attitude	Place of anthrax vaccination (n = 495)			
	Home	377	76.16	72.1–79.8
	Veterinary hospital	82	16.56	13.4–20.2
	Animal health check-up camps	36	7.28	5.2–10.0
	Misconceptions and challenges regarding anthrax vaccination (n = 495)			
	Productivity of animals will be hampered	90	18.19	14.9–21.9
	Vaccination is costly	86	17.38	14.2–21.0
	Livestock inspector is not coming home	61	12.32	9.6–15.6
	Animals become weak after vaccination	108	21.81	18.3–25.7
	None	150	30.30	26.3–34.6
	Respondents’ reaction to suspected anthrax cases in animals (n = 541)			
	Inform relevant authority	398	73.57	69.6–77.1
	Traditional methods	18	3.33	2.0–5.3
	Do not report anyone	125	23.1	19.6–26.9
Practices	Vaccinated their livestock animals against anthrax (n = 1907)			
	Yes	406	21.29	19.5–23.2
	No	1501	78.71	76.7–80.5
	Period of conduction of anthrax vaccination of animals (n = 406)			
	Less than 6 months	245	60.35	55.4–65.1
	6 months–1 year	117	28.81	24.5–33.5
	1–2 years	23	5.67	3.7–8.5
	More than 2 years	21	5.17	3.3–7.9
	Was the anthrax vaccination free of cost (n = 406)			
	Yes	162	39.90	35.1–44.8
	No	244	60.10	55.1–64.8
	Respondent’s meat consuming habit (n = 2670)			
	Yes	2393	89.63	88.3–90.7
	No	277	10.37	9.3–11.6
	Type of meat the respondents consume^#^ (n = 2393)			
	Beef	353	14.75	13.4–16.2
	Pig	135	5.64	4.7–6.6
	Sheep	2204	92.1	90.9–93.1
	Goat	1810	75.64	73.8–77.3
	Respondent’s consuming animal blood in their diet (n = 2393)			
	Yes	599	25.03	23.3–26.8
	No	1794	74.97	73.1–76.6
	Respondents managing dead bodies of livestock animals (n = 1907)			
	Burial	1591	83.43	81.6–85.1
	Throw them away	129	6.77	5.7–8.0
	Distribute among villagers	55	2.88	2.2–3.7
	Selling the carcass	51	2.67	2.0–3.5
	Consume the meat	81	4.25	3.4–5.3

^#^Multiple choices were noted

The odds ratio of both unadjusted and adjusted with their 95% confidence intervals (CIs) were used to assess the association as in Table 4. People with the age group 30–39 years had 1.4 times higher odds of possessing knowledge about anthrax compared to the people of the reference age group. Males were more likely to possess the knowledge of anthrax than females which might be due to the livestock handling predominantly done by them. Compared to the participants who received no formal education, those who had received formal education were two times more likely to possess the knowledge of anthrax. Participants who had more than 10 years of experience in handling livestock were 1.4 times more probable of possessing the knowledge of anthrax than others who had less than 10 years of experience.

**Table 4:** Bivariate and multivariate logistic regression analysis output of factors associated with knowledge of anthrax

Characteristic	Comparison	Knowledge of anthrax	Total	Unadjusted odds ratio	95% C.I.	p-value	Adjusted odds ratio	95% C.I.	p-value
Age	18–29	138	714	Reference					
	30–39	162	633	1.43	1.11–1.85	< 0.01	1.48	1.08–2.03	0.02
	40–49	105	542	1	0.75–1.33	0.98	0.96	0.69–1.42	0.96
	50–59	73	397	0.94	0.68–1.28	0.7	1.07	0.72–1.57	0.73
	60 & above	63	384	0.81	0.59–1.13	0.23	0.98	0.65–1.49	0.95
Gender	Female	60	634	Reference					
	Male	481	2036	2.95	2.22–3.93	< 0.01	2.20	1.50–3.22	< 0.01
Education	Illiterate	191	1432	Reference					
	Literate	350	1238	2.56	2.10–3.11	< 0.01	2.17	1.68–2.79	< 0.01
Occupation	Non-agriculture	243	1260	Reference					
	Agriculture	298	1410	1.12	0.92–1.35	0.23	1.09	0.85–1.39	0.47
Livestock	Absent	138	763	Reference					
	Present	403	1907	1.21	0.97–1.50	0.07	1.17	0.93–1.47	0.18
Experience of handling livestock	Less than 10 years	103	624	Reference					
	More than 10 years	300	1283	1.54	1.20–1.97	0.01	1.49	1.14–1.94	< 0.01
Meat consumption	No	31	277	Reference					
	Yes	510	2393	2.14	1.46–3.16	< 0.01	1.79	1.13–2.82	0.01

## Discussion

The study provides preliminary baseline information regarding the knowledge, attitude and practices of anthrax among the people of Koraput district. In our study, socio-demographic characteristics from the community were significantly associated with the knowledge of anthrax. Many communities in the district have very low levels of understanding of human and animal anthrax disease, which leads them to the risk of anthrax exposure as well as underreporting of the cases to the health officials. There are some harmful practices which include eating livestock blood, eating dead animals’ meat, distributing or selling carcasses to local people and even throwing carcasses anywhere in the field. Such occurrences may lead to environmental contamination by anthrax spores [14]. Once the spores are released into the environment, they can remain viable for a period of 90 years or more and can be a source of future outbreaks [15]. The attention of authorities towards anthrax is sought when outbreaks happen in the community, making people sick or dead after consuming infected or uninspected meat [16]. Almost 71% of the population owns livestock of which 96% have cattle/buffalo, which indicates that the community is highly dependent on cattle for livelihood. This could be one of the major reasons for frequent outbreaks in cattle than any other livestock in Koraput [17]. Majority of livestock visit the forest for grazing which can be another factor of ingesting spores and a possible risk factor for anthrax disease outbreaks among animals. Several studies have hypothesized the seasonal variations or climate conditions for anthrax disease outbreaks, and there is strong evidence regarding their association [18–20]. Around 21.2% of livestock owners have vaccinated their animals against anthrax disease which shows a lack of anthrax vaccination knowledge and practice in the community. Veterinarians contribute a major role in building and implementing disease control programs in livestock. The annual vaccination of livestock against anthrax significantly lowers the odds of acquiring anthrax than those that were never vaccinated [21]. Non-livestock owners had less knowledge about anthrax disease than livestock owners, as it was expected that the disease occurs mainly in livestock animals but it was not significantly associated. The Government is providing financial assistance for the burial of dead animals to the livestock owners but more than 98% of the livestock owners are unaware of the incentive as per our study. Livestock owners had indigenous knowledge of various diseases affecting their livestock but awareness of anthrax disease, vaccination of animals against anthrax and the government incentive for burial of dead animals was lacking in most cases which must be done by the local governmental organizations or veterinary department. The education of livestock owners for the handling of anthrax suspect cases or dead livestock animals is very important by training them on the proper burial of animals and not to cut such carcasses or consume them. Anthrax is a neglected zoonotic disease that is often underreported from its actual prevalence [22]. Establishing a surveillance network for reporting anthrax cases in endemic districts can be beneficial in preventing outbreaks of the disease. The findings from the study could open the doors for future contributions, research efforts and also can be used to optimize prevention and control strategies, including vaccination of livestock and educational campaigns of livestock owners.

## Conclusion

This study highlights substantial knowledge and practice gaps on anthrax in the community which indicates a need for improvement in these areas. Several risk practices were identified such as consumption, distribution and trading of dead animal meat which could be the potential factors in the transmission of anthrax from animal to human. A surveillance system for early case detection and proper education among the community regarding anthrax is essential for the timely detection, prevention and control of outbreaks. Immediate measures should be taken to maximize the coverage of anthrax vaccination among the livestock of the district. Active inter-departmental coordination by adopting the One Health approach in endemic regions is a potential method to address these gaps.

## Supplementary Information

**Additional file 1.** Block-wise sample collection distribution.

## Data Availability

Raw data will be available on request to the corresponding author.
