# Supplementary material for: Assessment of socio-behavioural correlates and risk perceptions regarding anthrax disease in tribal communities of Odisha, Eastern India
Source: BMC Infect Dis. 2022 Jan 15;22:53. doi: 10.1186/s12879-022-07035-9 (PMC8760694; doi:10.1186/s12879-022-07035-9)
Supplement: Supplementary file 1 — Additional file 1. Block-wise sample collection distribution. [file 12879_2022_7035_MOESM1_ESM.docx]

**ANNEXURE -1**

| **Name of Blocks** | **Proportion allocation**  **according to 2011 Census** | **Participants required per village** | **Villages per block** | **Participants required per block** |
| --- | --- | --- | --- | --- |
| Pottangi | 5% (130) | 17 | 8 | 136 |
| Dasamantapur | 6% (156) | 20 | 8 | 160 |
| Kotpad | 8% (208) | 26 | 8 | 208 |
| Boriguma | 12% (312) | 39 | 8 | 312 |
| Lakshmipur | 5% (130) | 17 | 8 | 136 |
| Bandhugaon | 4.5% (117) | 15 | 8 | 120 |
| Narayanpatana | 4.5% (117) | 15 | 8 | 120 |
| Semiliguda | 6% (156) | 20 | 8 | 160 |
| Koraput | 9% (234) | 30 | 8 | 240 |
| Jeypur | 15% (390) | 49 | 8 | 392 |
| Kundura | 5.5% (143) | 18 | 8 | 144 |
| Boipariguda | 8% (208) | 26 | 8 | 208 |
| Lamtaput | 4.5% (117) | 15 | 8 | 120 |
| Nandapur | 7% (182) | 23 | 8 | 184 |
| **TOTAL** | **100 % (2608)** |  |  | **2640** |

**ANNEXURE -2**

| **DISTRICT** | **BLOCK** | **GRAM PANCHAYAT** | **VILLAGES** |
| --- | --- | --- | --- |
| **KORAPUT** | **Pottangi** | Chandaka | Gugaguda, Chandaka, Baraja, Pangiguda |
|  |  | Maliput | Sindhei, Sorgiguda, Maliput, Sepaiput |
|  | **Dasmanthpur** | Lulla | Panjaraput, Kemili, Lulla, Gulimusha |
|  |  | Pindapadar | Maruar, Sorispadar, Baragacha, Birighat |
|  | **Laxmipur** | Laxmipur | Kesakapidi, Daliamba, Nisar, Ranjitguda |
|  |  | Toyaput | Toyaput, Raisili, Dumuripadar, Uskabhata |
|  | **Bandhugaon** | Alamanda | Keshabhadra, Idumgumabalsa, Barabankeri, Hatigeda |
|  |  | Bandhugaon | Turukpeta, Singhiput, Dasini, Gotiput |
|  | **Koraput** | Dumuripadar | Dumuripadar, Kerengam, Hatimunda, Pendajam |
|  |  | Mastiput | Tolla, Dangari, Bandiguda, Hiridiput |
|  | **Narayanapatna** | Balipeta | Panabari, Gotiguda, Dumusil, Bejuguda |
|  |  | Tentulipadar | Kumarsil, Rangapani, Kamalapodar, Tentulipadar |
|  | **Nandapur** | Malibelagaon | Kujel, Baratankua, Chheliamunda, Gulpandi |
|  |  | Goluru | Kuda, Podeising, Damanga, Goluru |
|  | **Semiliguda** | Dudhari | Dudhari, Sundhiput, Barokutuni, Putsil |
|  |  | Kunduli | Pungar, Kunduli, Sirimunda |
|  |  | Pakhijola | Pakhijola |
|  | **Lamtaput** | Godihanjar | Anungu, Badakichaba, Godhihanjar, Bangurupoda |
|  |  | Jalahanjar | Tukum, Pipalaput, Podapodar, Mukhiput |
|  | **Kotpad** | Chatralla | Mokagam, Dumajodi, Chatralla, Mundaguda |
|  |  | Murtahandi | Monchahandi, Sindhigam, Ambogam, Murtahandi |
|  | **Boipariguda** | Doraguda | Minarbali, Doraguda, Banaguda, Bergan |
|  |  | Kathpada | Kenduput, Basiniguda, Kathpada, Talapadar |
|  | **Borigumma** | Dengapadar | Dengapadar, Kerkenda, Patigan, Porsola |
|  |  | Kamara | Porli, Kamara, Bhatigam, Mundiguda |
|  | **Jeypore** | Konga | Konga, Khodopa |
|  |  | Ekomba | Ekomba, Gaganapur |
|  |  | Hadia | Hadia, Putra, Bhatra, Khairamundi |
|  | **Kundra** | Bagderi | Kanaguda, Jamukonadi, Dangarapalli, Bagderi |
|  |  | Karmit | Karmit, Jabapadara, Kadalimunda, Kondajodi |
